# *ATM* rs189037 (G > A) polymorphism increased the risk of cancer: an updated meta-analysis

**DOI:** 10.1186/s12881-019-0760-8

**Published:** 2019-02-01

**Authors:** Zhi-liang Zhao, Lu Xia, Cong Zhao, Jun Yao

**Affiliations:** 1 Hospital Office, Chengdu First People’s Hospital, Chengdu, 610000 Sichuan Province People’s Republic of China; 2 Department of Anesthesia, Critical Care and Pain Medicine, Massachusetts General Hospital, Harvard Medical School, Charlestown, MA USA; 3 Department of Rehabilitation, Chengdu First People’s Hospital, Chengdu, 610000 Sichuan Province People’s Republic of China; 4 Department of Gastroenterology, Chengdu First People’s Hospital, Chengdu, 610000 Sichuan Province People’s Republic of China; 5 School of Forensic Medicine, China Medical University, No. 77 Puhe Road, Shenbei New District, Shenyang, 110122 China

**Keywords:** ATM, Cancer, Meta-analysis, Gene polymorphism

## Abstract

**Background:**

Rs189037 (G > A) is a functional single nucleotide polymorphism (SNP) in the Ataxia-telangiectasia mutated (ATM) gene that may be associated with the risk of cancer. We performed a meta-analysis to determine whether rs189037 polymorphism influences the occurrence of cancer and examined the relationship between this SNP and the etiology of cancer.

**Methods:**

Case-control studies were retrieved from literature databases in accordance with established inclusion criteria. Odds ratios (ORs) and 95% confidence intervals (CIs) were calculated to evaluate the strength of the association between rs189037 and cancer. Subgroup analysis and sensitivity analysis also were performed.

**Results:**

After inclusion criteria were met, fifteen studies—comprising 8660 patients with cancer (cases) and 9259 controls—were included in this meta-analysis. Summary results indicated that an association was found between rs189037 and cancer risk. In the dominant model, the pooled OR using a random effects model was 1.207 (95% CI, 1.090–1.337; *P* < 0.001). The A allele of rs189037 increased the risk of lung cancer, breast cancer, and oral cancer. Results of subgroup analysis by ethnicity indicated that the SNP was associated with the risk of cancer among East Asian and Latino, but not Caucasian.

**Conclusions:**

Results of this meta-analysis suggest that rs189037 is associated with the occurrence of lung cancer, breast cancer, and oral cancer as the risk factor. These data provide possible avenues for future case-control studies related to cancer.

## Background

The occurrence of cancer is increasing because of the population aging, smoking, physical inactivity, et al [1]. It is a cellular abnormality, uncontrolled growth caused by numerous damages or mutations in the genetic material due to hereditary or environmental factors, which is immune to many signals that control cell growth and death [2]. The genetic factors takes more proportion on the causation of cancer than the lifestyle or environmental factors [3]. Many candidate genes or variations have been identified to contribute to the susceptibility of the cancer.

Ataxia-telangiectasia mutated (ATM) gene is located on the chromosome 11q22–23 with the full length 150 kb [4]. It comprises 66 exons and encodes a 12 kb transcript. The encoded protein belongs to the PI3/PI4-kinase family. As a Ser/Thr protein kinase, ATM protein plays an important role in DNA damage-induced signaling and initiation of cell cycle check-point signaling by phosphorylating [5]. After activated by DNA double strand breaks, it can be involved in recognizing broken or damaged DNA stands and assisting DNA repair by recruiting enzymes to recover the damaged strands [6]. Three deleterious missense variants of ATM gene were associated with an increased risk of cancer [7]. Moreover, ATM mutation and ATM protein loss included characteristics of old age, distal location of tumor, large tumor size, and histologic intestinal type in the human gastric cancer tissue [5]. The missense variants in ATM gene were also associated with the prostate cancer predisposition [8]. The loss of ATM function can give rise to ataxia telangiectasia, a pleiotropic disease with the whose hallmarks, such as neurodegeneration, cancer-proneness, premature aging, radio-sensitivity, et al [9]. It can control genome stability, modulate oxidative stress response, autophagy, and cancer stem cell survival as tumor suppressor gene [10].

The variation of ATM gene can affect the normal function of the protein and increase the risk of cancer. Rs189037 (G > A) is located at the 5’UTR of ATM gene and is one of the critical polymorphism that may be related to the occurrence of different cancers and tumor diffusing capacity [11–15]. However, no consistent conclusion has been determined, and there remains discord between the findings in the literature, which may be attributable to a number of factors varying between studies including the types of cancer, the sample sizes, the genetic backgrounds of study subjects, and the potential presence of confounding bias [16].

When there is considerable variation in the results of studies on medical topics that have been studied extensively, meta-analysis can be used as a method to identify a common effect [17]. Such an analysis was conducted by Kang et al. (2014) to assess whether the ATM rs189037 polymorphism was associated with the risk of papillary thyroid carcinoma [18]. But only one case-control study was focused on rs189037. Bhowmik et al. analyzed the association of rs189037 with the risk of lung cancer and head and neck cancer in 2015 [19]. A total of 9 case-control studies were considered for this quantitative analysis. The third 2017 meta-analysis including ten case-control studies (4731 cases and 5142 controls) also reported the association between rs189037 and lung cancer susceptibility [12]. It seems superfluous to perform the meta-analysis of rs189037 and its association with cancer risk, whereas that the two latest meta-analyses only focused on the lung cancer and there are additional studies reporting its role in the other cancer types, such as breast cancer, papillary thyroid carcinoma, leukemia [14, 15, 20]. Therefore, we have performed a new meta-analysis of the ATM rs189037 polymorphism and the risk of different cancer types that includes more recent research.

## Methods

### Identification of relevant studies

We performed a literature search of three online literature databases (PubMed, Web of Science and Embase) to screen and identify available studies to be included in the meta-analysis. The keywords that were used are as follows: ATM, ataxia-telangiectasia mutated, rs189037, and cancer. Additionally, other possible studies were screened from the reference lists of included studies and relevant reviews.

The inclusion criteria were as follows: [1] the study were designed as case-control; [2] the cases in the identified studies were cancer patients; and [3] the studies reported the frequencies of ATM alleles and/or genotypes. When authors published multiple articles using the same or overlapping datasets, we selected the most recent study for inclusion. Exclusion criteria included the omission of healthy controls or the duplication of earlier research. In the event that inclusion data – including allele frequency, genotype or another sample characteristic – were not present in a report, we contacted the authors by email for the relevant information.

### Data extraction

Two investigators (Zhi-liang Zhao and Lu Xia) independently extracted the data from each eligible publication, including the last name of the first author, the year of publication, the geographic region, the genotyping method, the sample size, and the number of genotypes reported for both cases and controls. In addition, to determine the contributions of underlying characteristics on the findings of the included reports, we also extracted data regarding patient ethnicities, sources of controls, and types of cancer.

### Quality assessment

The quality of the included studies was assessed by the Newcastle Ottawa Scale (NOS) (http://www.ohri.ca/programs/clinical_epidemiology/default.asp). The scores of five or more (maximum of nine) were considered “high quality”, while the studies with the scores under five were regarded as “low quality”.

### Statistical analysis

The Hardy-Weinberg equilibrium of control genotypes was calculated using a χ^2^ test. The strength of the association of rs189037 and cancer was evaluated with ratios (ORs) and 95% confidence intervals (CIs). A random effects model to resolve inter-study heterogeneity was used to calculate pooled estimates of the ORs and 95% CIs among the included studies [21].

Three genetic models (allele contrast model, dominant model, and recessive model) were used to measure the overall pooled ORs. As described in the previous study, OR_1_ (GG vs. AA), OR_2_ (GG vs. GA), and OR_3_ (GA vs. AA) were compared, with the definition of A as the risk allele [17]. If OR_1_ = OR_3_ ≠ 1 and OR_2_ = 1, then a recessive model was selected. If OR_1_ = OR_2_ ≠ 1 and OR_3_ = 1, then a dominant model was selected. If OR_2_ = 1/OR_3_ ≠ 1 and OR_1_ = 1, then a complete overdominant model was selected. If OR_1_ > OR_2_ > 1 and OR_1_ > OR_3_ > 1 (or OR_1_ < OR_2_ < 1 and OR_1_ < OR_3_ < 1), then a codominant model was selected [22, 23].

We evaluated the degree of inter-study heterogeneity using a Q statistic [24, 25], where *P* > 0.05 was defined as an absence of heterogeneity [26]. We performed subgroup analysis for ethnicity (i.e., Caucasian, East Asian, etc.) and source of controls (i.e., hospital- or population-based).

We evaluated whether a single study potentially influenced the pooled effect size by means of sensitivity analysis. Specifically, we omitted each study from the meta-analysis in turn and subsequently evaluated whether any significant alterations were made to the pooled effect size.

Publication bias was investigated by using funnel plots generated for each study in which the standard error of log(OR) was plotted against the log(OR). Possible publication bias was determined when the plot was asymmetric, in which case an Egger test was used to determine degree of asymmetry, with *P* < 0.05 indicating publication bias [27].

All the statistical calculations were performed by Stata version 10.0 (Stata Corp., College Station, TX).

## Results

We searched the database and identified 219 articles. According to the established inclusion criteria, a total of 15 publications were finally screened and included in our meta-analysis [13–15, 20, 28–38]. We collected 15 case-control studies, which contained 8660 patients with cancer (i.e., cases) and 9259 unaffected participants (i.e., controls). The individuals with the different genetic backgrounds were included (e.g., East Asian, Latino, and Caucasian). The main characteristics of the included studies were summarized in Table 1. Based on the results of the NOS scale, 12 studies were regarded as high quality and 3 studies were regarded as low quality. The genotype and allele frequencies of rs189037 SNP and HWE in controls were presented in Table 2. Of the 15 studies, no study deviated significantly from HWE.

**Table 1:** Baseline characteristics of qualified studies in this meta-analysis

Author	Year	Region	Ethnicity	Controls source	Type of cancer	Genotyping method	Case/control	Male(case/control)	NOS scores
Kim	2006	Korea	East Asian	hospital-based	lung cancer	SNaPShot assay	616/616	483/483	4
Wang	2010	Taiwan	East Asian	hospital-based	breast cancer	PCR-RFLP	1232/1232	0/0	5
Bau	2010	Taiwan	East Asian	hospital-based	oral cancer	PCR-RFLP	620/620	586/582	5
Lo	2010	Taiwan	East Asian	hospital-based	lung cancer	MassARRAY	730/730	384/384	5
Wang	2011	Taiwan	East Asian	hospital-based	leukemia	PCR-RFLP	266/266	148/148	5
Xu	2012	Brazil	Latino	hospital-based	differentiated thyroid cancer	TaqMan	592/885	146/379	6
Hsia	2013	Taiwan	East Asian	hospital-based	lung cancer	PCR-RFLP	358/716	254/488	5
Zhao	2013	China	East Asian	hospital-based	glioma	TaqMan	384/384	222/217	6
Damiola	2013	Belarus	Caucasian	population-based	papillary thyroid carcinoma	Illumina GoldenGate Genotyping Assay	83/324	35/127	7
Gu	2014	China	East Asian	hospital-based	papillary thyroid carcinoma	MALDI-TOF-MS	358/360	109/115	4
Liu	2014	China	East Asian	population-based	lung cancer	TaqMan	852/852	485/490	5
Shen	2014	China	East Asian	hospital-based	lung cancer	TaqMan	487/516	0/0	5
Song	2014	Korea	East Asian	hospital-based	papillary thyroid carcinoma	TaqMan	437/184	93/51	6
Yue	2018	China	East Asian	hospital-based	breast cancer	ligase detection reaction method	524/518	0/0	4
Wang	2018	China	East Asian	hospital-based	colorectal cancer	TaqMan	1121/1056	631/561	5

**Table 2:** Distribution of genotype and allele frequencies of the *ATM* rs189037 polymorphism

	Genotype distribution							Allele frequency			
	Cases, n			Controls, n				Cases, %		Controls, %	
Author	GG	GA	AA	GG	GA	AA	*P*_HWE_	G	A	G	A
Kim	190	316	105	195	306	113	0.7130	56.96	43.04	56.68	43.32
Wang	428	580	224	474	567	191	0.3210	58.28	41.72	61.49	38.51
Bau	181	277	162	239	285	96	0.4704	51.53	48.47	61.53	38.47
Lo	238	345	145	239	354	124	0.7173	56.39	43.61	58.02	41.98
Wang	89	128	49	106	119	41	0.4295	57.52	42.48	62.22	37.78
Xu	375^a^		215	606^a^		277	–	–	–	–	–
Hsia	118	176	64	255	339	122	0.6068	57.54	42.46	59.29	40.71
Zhao	140	186	58	125	203	56	0.0697	60.68	39.32	58.98	41.02
Damiola	13	32	23	35	106	60	0.3121	42.65	57.35	43.78	56.22
Gu	90	196	69	102	189	69	0.2638	52.96	47.04	54.58	45.42
Liu	217	435	200	264	434	154	0.2927	51.00	49.00	56.46	43.54
Shen	148	240	99	152	272	92	0.1186	55.03	44.97	55.81	44.19
Song	134	211	83	56	84	42	0.3352	55.96	44.04	53.85	46.15
Yue	166	262	96	196	258	64	0.1371	56.68	43.32	62.74	37.26
Wang	336	543	227	362	491	191	0.2797	54.93	45.07	58.19	41.81

*Abbreviation*: *P*_HWE_ represents the *P* value of Hardy-Weinberg equilibrium test in the genotype distribution of controls

^a^represents the number of GG + GA

### Heterogeneity detection and pooled analysis

**Fig. 1:**
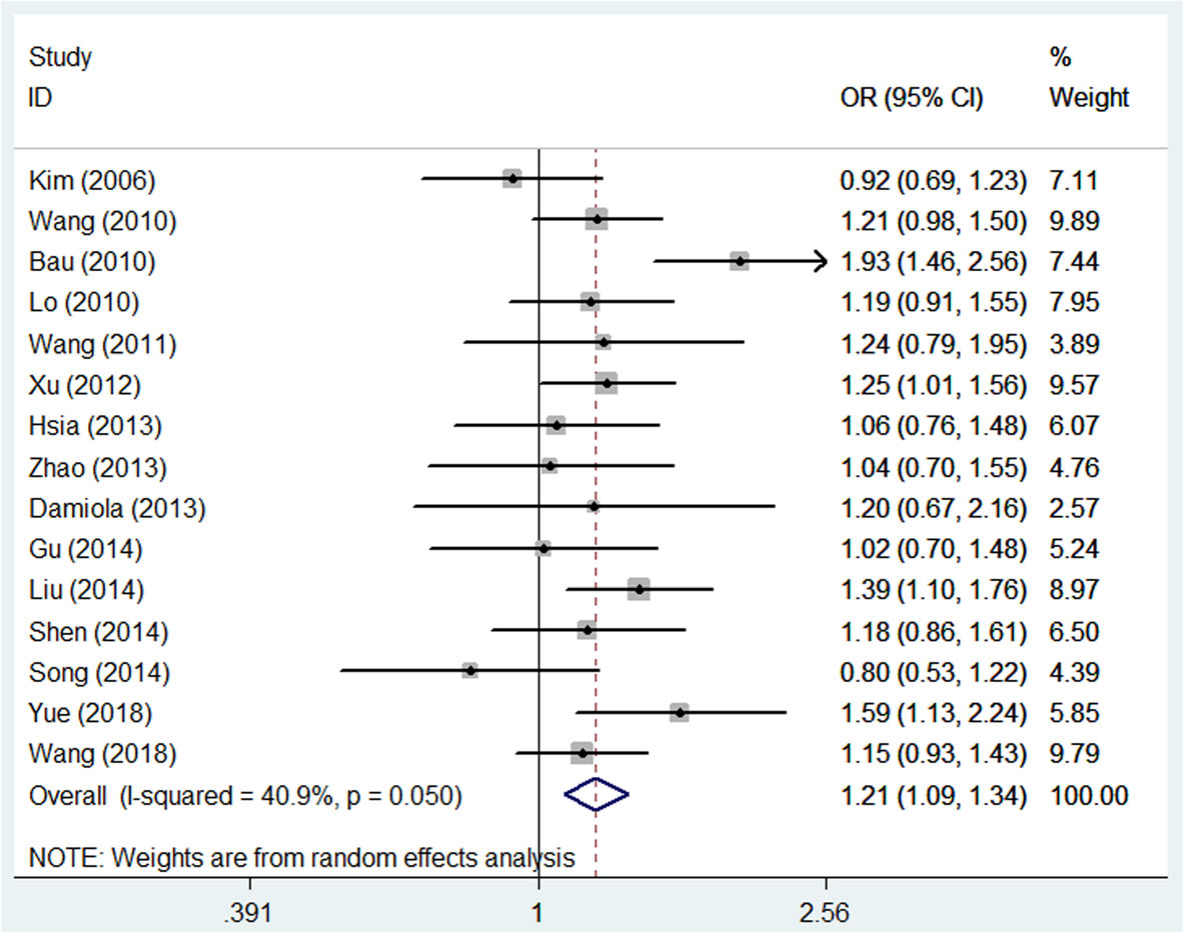
Forest plot of the association between the rs189037 polymorphism of *ATM* and cancer in the dominant genetic model (GG + GA vs. AA)

The association between the rs189037 polymorphism and cancer risk was evaluated using pooled ORs (with 95% CIs) under dominant, recessive, homozygous codominant, heterozygous codominant and allele contrast genetic models (Fig. 1, Table 3). Finally, we selected the dominant model to perform the pooled analysis [22, 39]. The pooled results showed that rs189037 polymorphism was associated with cancer risk. In the dominant model, the summary OR generated by a random effects model was 1.207 (95% CI, 1.090–1.337; *P* < 0.001). The A allele of rs189037 increased the risk of cancer. Results of subgroup analysis by ethnicity indicated that the SNP was associated with the risk of cancer among East Asian and Latino, but not Caucasian (Table 4). Moreover, the association between rs189037 and cancer was observed in subgroup analysis according to the source of controls (hospital based and population-based). Additionally, we also performed the subgroup analysis by the type of cancer. The results showed that rs189037 increased the occurrence of lung cancer, breast cancer, and oral cancer, but not leukemia, thyroid carcinoma, glioma, and colorectal cancer (Table 4).

**Table 3:** Summarized ORs with 95% CIs for the association of *ATM* rs189037 polymorphism with cancer

Polymorphism	Genetic model	n	Statistical model	OR	95% CI	*p*_z_	I^2^(%)	*p*_h_	*p*_e_
Rs189037									
	Allele contrast	14	Random	1.123	1.049–1.202	0.001	53.5	0.009	0.337
	Homozygous codominant	14	Random	1.267	1.105–1.454	0.001	52.0	0.012	0.308
	Heterozygous codominant	14	Random	1.159	1.049–1.281	0.004	23.5	0.200	0.624
	Dominant	15	Random	1.207	1.090–1.337	< 0.001	40.9	0.050	0.415
	Recessive	14	Random	1.151	1.061–1.247	0.001	27.5	0.160	0.272

n, the number of studies; *p*_z_, *P* value for association test; *p*_h_, *p* value for heterogeneity test; *p*_e_, *p* value for publication bias test

**Table 4:** Stratified analysis of the association of *ATM* polymorphisms with cancer under dominant model

Subgroup analysis	Rs189037					
	n	OR	95% CI	*p*_z_	I^2^(%)	*p*_h_
Overall	15	1.207	1.090–1.337	< 0.001	40.9	0.050
Ethnicity						
East Asian	13	1.200	1.067–1.350	0.002	49.2	0.023
Latino	1	1.254	1.007–1.563	0.043	–	–
Caucasian	1	1.201	0.668–2.159	0.540	–	–
Source of controls						
Hospital-based	13	1.189	1.061–1.332	0.003	46.3	0.034
Population-based	2	1.362	1.095–1.695	0.006	0.0	0.650
Type of cancer						
lung cancer	5	1.158	1.005–1.334	0.043	19.9	0.288
breast cancer	2	1.341	1.035–1.737	0.026	43.3	0.184
oral cancer	1	1.931	1.456–2.559	< 0.001	–	–
leukemia	1	1.239	0.786–1.953	0.355	–	–
thyroid carcinoma	4	1.094	0.897–1.335	0.374	20.3	0.288
glioma	1	1.042	0.700–1.551	0.839	–	–
colorectal cancer	1	1.153	0.931–1.429	0.192	–	–

n, the number of studies; *p*_z_, *p* value for association test; *p*_h_, *p* value for heterogeneity test

### Sensitivity analysis

We next sought to determine the contribution of individual studies to the pooled results via sensitivity analysis. To do this, we removed each study from the analysis, in turn, and then determined pooled ORs. We detected no significant changes between each of these analyses and the overall results of the meta-analysis, indicating that none of the included studies significantly altered the overall results. Therefore, our meta-analysis results are stable and reliable.

### Publication bias

**Fig. 2:**
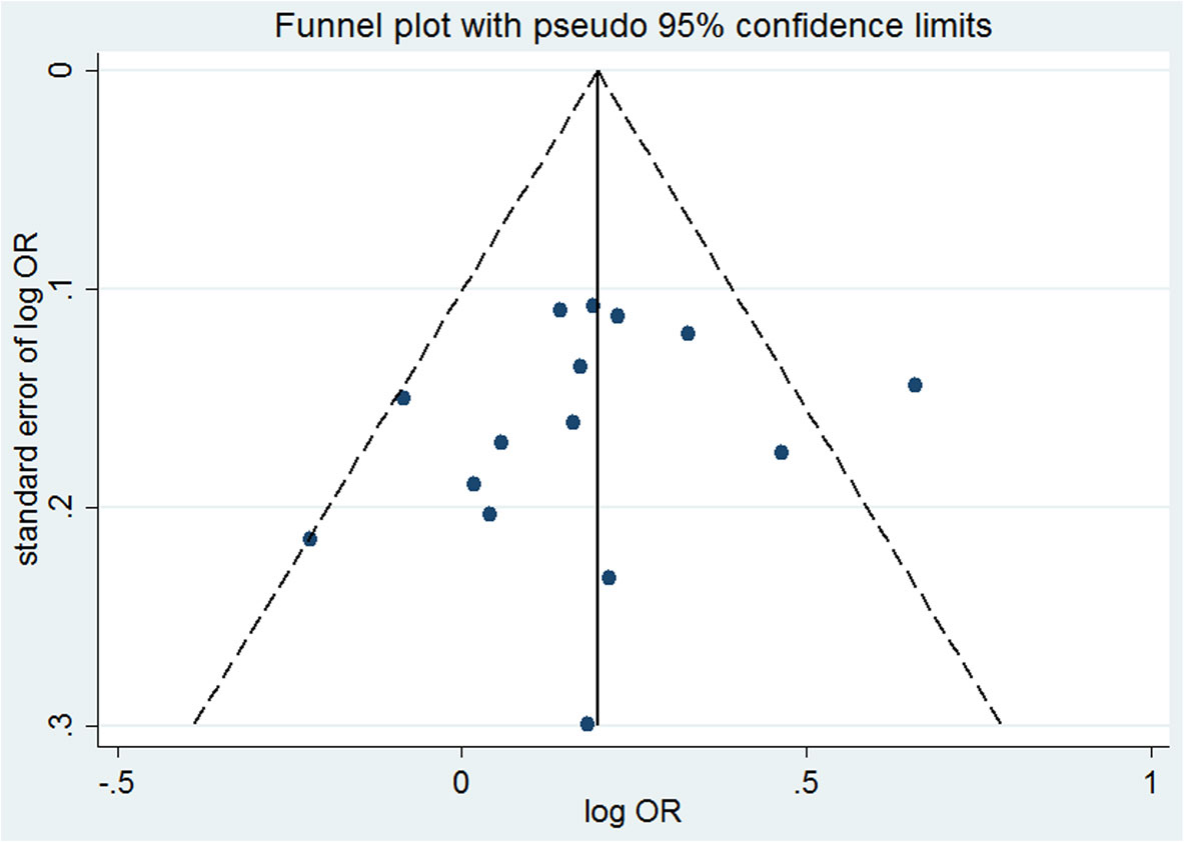
Funnel plot analysis depicting publication bias in the association between the rs189037 polymorphism of *ATM* and cancer

Publication bias was assessed by generating and analyzing a funnel plot (Fig. 2), and no significant effect of publication bias was detected (*P*_e_ = 0.415) (Table 3).

## Discussion

We explored the underlying relationship between rs189037 SNP of *ATM* gene and the occurrence of cancer using a meta-analysis that included 15 case-control studies (8660 cases and 9259 controls). The pooled results indicated that there was an association, and subgroup analysis by ethnicity and source of controls further investigated the distribution deviation between cases and controls.

Previously, three meta-analyses have reported the putative association between rs189037 and the occurrence of cancer [12, 18, 19]. Generally, our results were consistent with the previous studies. It seems that our meta-analysis is redundant, but there are some highlights compared with the previously published studies. Firstly, our analysis included the newly published studies since the previous meta-analyses were performed. A total of 15 studies were included, which could comprehensively represent rs189037 better compared with the previous meta-analyses. Additionally, the subgroup analyses were carried out by ethnicity, source of controls, and types of cancer to explore the potential origins of heterogeneity and to measure the study stability. Thus, to some degree, our meta-analysis could give a more accurate, comprehensive finding that there is an association between rs189037 SNP and lung cancer, breast cancer, and oral cancer, but not leukemia, thyroid carcinoma, glioma, and colorectal cancer.

However, the relatively small sample sizes of Latino and Caucasian populations limited our ability to isolate stable effects for these subgroups. Only one study reported the association of rs189037 with differentiated thyroid cancer in Latino including 592 cases and 885 controls [31]. For Caucasian, there is also just one study about the risk of papillary thyroid carcinoma including 83 cases and 324 controls [34]. Thus, we cannot obtain the comprehensive results of the association between rs189037 and cancer risk in Latino and Caucasian population because of the limited sample size.

Rs189037 is in the promoter region of *ATM* gene and markedly changes the folding architectures. The secondary structure of rs189037 G/A alleles was significant changed using RNAfold prediction [38]. It has been confirmed to be associated with carcinogenesis [38, 40]. The G allele of rs189037 SNP is an independent risk factor for radiation-induced pneumonitis in Chinese thoracic cancer patients [41]. Moreover, rs189037 and other polymorphism in DNA repair genes can serve as candidate prognostic markers of the survival of non-small-cell lung cancer patinets [42]. The combined analysis showed that this SNP was associated with the poor prognosis. In addition, Piaceri et al. reported that the rs189037 was associated with the longevity in Italian centenarians [43]. Taken into account that the A allele of rs89037 increased the risk of cancer in our meta-analysis, we need to do more efforts to explore its influence on the expression of ATM protein.

However, there are some potential limitations in our current analysis. Firstly, the significant heterogeneity were detected in summary and subgroup analyses. Though the subgroup analysis was used to explore the possible origins of heterogeneity, no single factor could fully explain the heterogeneity. When the subgroup analysis was performed by the cancer types, the results showed that rs189037 increased the occurrence of lung cancer, breast cancer, and oral cancer, but not leukemia, thyroid carcinoma, glioma, and colorectal cancer. Clearly, the role of rs189037 polymorphism was influenced by cancer types. Thus, more cancer types need to be included and assessed in the future in order to comprehensively explore the effect of rs189037 in the cancer risk. Secondly, we did not analysis the gene-gene interactions and epigenetic, which were the influence factors of the cancer. Smoking, physical activity, and emotional state are also involved in the occurrence of cancer. Thirdly, just one SNP in *ATM* gene was analyzed and its information was limited. The occurrence of the cancer is usually thought to involve the multiple genes and their interactions.

## Conclusions

Our study showed that there was an association between the rs189037 in *ATM* gene and lung cancer, breast cancer, and oral cancer. The studies containing different ethnicity populations need to validate the findings of this meta-analysis and to ascertain the epigenetic mechanisms and environmental influences that contribute to the risk of cancer.

## Abbreviations

ATM: Ataxia-telangiectasia mutated
CIs: Confidence interval
HWE: Hardy-Weinberg equilibrium
ORs: Odds ratios
SNP: Single nucleotide polymorphism
